# Prevalence of food allergens sensitization and food allergies in a group of allergic Honduran children

**DOI:** 10.1186/s13223-018-0245-x

**Published:** 2018-06-18

**Authors:** Victoria Alejandra Gonzales-González, Adolfo Martin Díaz, Karla Fernández, María Félix Rivera

**Affiliations:** 1 Division of Epidemiology, Universidad Nacional Autonoma de Honduras, Tegucigalpa, Francisco Morazán, Honduras; 2 Division of Pediatric Allergy and Immunology, Hospital María de Especialidades Pediátricas, Tegucigalpa, Francisco Morazán, Honduras; 3 Division of Epidemiology, Facultad de Ciencias Medicas, Universidad Nacional Autonoma de Honduras, Tegucigalpa, Francisco Morazán, Honduras

**Keywords:** Sensitization, Skin prick test, Allergens, Children, Allergy

## Abstract

**Background:**

Food allergy is a public health problem that has increased in the last decade. Despite the increasing rates in children, quality data on the burden of these diseases is lacking particularly in developing countries. Honduras has no studies in pediatric patients.

**Objectives:**

The objective of this research was to identify the most common sensitization patterns to food through epicutaneous skin testing and food allergy rates in children and their correlation with common allergic diseases in a group of patients from Hospital of Pediatrics Maria.

**Methods:**

Cross-sectional retrospective, descriptive study in which records and database of all allergic patients in the immunology outpatient clinic from Hospital of Pediatrics Maria were reviewed between the periods of January 2015 through June 2016.

**Results:**

A total of 365 children were analyzed, the age of participants were in the range from 1 to 18 years, with an average of 9.8 years. Sensitization to food allergens were found in 23, and 58.3% were poly-sensitized. The most common food allergens that patients were sensitized to: milk 9.0%, eggs 6.9%, peanut 4.9% and pork meat 4.4%. Food allergy was confirmed via oral food challenged in 9.3% of the patients. The most frequent food allergies found were: cow’s milk allergy 6%, hen’s egg allergy 5.2% and wheat allergy 1.9%.

**Conclusions:**

Milk and egg were the most common a food allergens found in the population studied. Most of the patients were found to be poly-sensitized. The frequent food allergies confirmed via oral food challenge were cow’s milk allergy, hen’s egg allergy and wheat allergy.

## Background

Sensitization, or the presence of allergic antibody, is a pre-requisite for development of an allergic response to an allergen. Patterns of sensitization to environmental and food allergens have previously been studied in children in an effort to better understand allergy development [1, 2]. A reaction to a food allergen can cause a wide range of clinical responses, ranging from urticaria to anaphylaxis, the most severe form of allergic reaction [3]. The current standard of care is based on identification and strict avoidance of triggering foods [4, 5]. If criteria for anaphylaxis are met, prompt administration of epinephrine is required [6].

Food allergy rates vary by age, local diet, and many other factors [7], however eight types of food account for over 90% of allergic reactions in affected individuals: milk, eggs, peanuts, tree nuts, fish, shellfish, soy, and wheat [8]. The evaluation of a child with suspected food allergy requires a detailed history and physical examination, and confirmatory tests, such as skin prick tests (SPT) and/or serum-specific IgE testing to foods (ImmunoCAP^®^). In some cases, oral food challenges (OFC) may also be required [9].

The prevalence of confirmed FA based on confirmatory tests is lower than perceived allergy, which is based on self-report. Diagnostic cut-off values for SPT have improved the diagnosis of food allergy and thereby reduced the need to perform oral food challenges [10]. However, it is a method prone to influence by a variety of factors that can add to the overall margin of error, with the potential to significantly alter test outcomes and adversely influence both the accuracy of diagnosis and the effectiveness of the subsequent immunotherapy regimens [11]. The specificity and sensitivity of skin testing is individually highly variable depending on age, body mass, and skin barrier status. In atopic inflammation skin testing gives more false positive results. Smaller skin area and strain limits prick testing in small children [12]. Other factors such as medication with antihistamines, certain antidepressants, calcineurin inhibitors or dermographism can alter the results [13].

Only one study in 50 adults have been done in Honduras by Sanchez et al. they reported a prevalence of 6% chocolate, sea food and corn, 4% for fish, peanut, beef and tomato and 2% for milk and chicken [14]. No further studies had been completed in children in this country.

The objective of this research was to identify the most common sensitizations through skin prick test and food allergy rates in children and their correlation with common allergic diseases in the group of patients from Hospital of Pediatrics Maria.

## Subject and methods

Cross-sectional retrospective study in which records and database of all allergic patients in the immunology outpatient clinic from Hospital of Pediatrics Maria were reviewed between the periods of January 2015 through June 2016. 365 files were reviewed. The diagnosis of allergic diseases was established according to the GINA guidelines for asthma, ARIA for rhinitis, Hanif and Rajka for diagnoses and SCORAD for assessing the severity of atopic dermatitis [15]. Urticaria and conjunctivitis were also included. Patients meeting the criteria of two or more allergic diseases were considered as multisystemic.

We used the skin prick test method for standard procedure with commercial extracts (ALK-Abello, Madrid, Spain) for 14 food products and OFC for diagnoses of food allergy. The food panel includes: milk (cows milk allergy and casein), soy, egg yolk, egg white, peanut, beef, pork, chicken, fish, wheat, rye, oats and banana. Food panel was picked according to the common Honduran diet and according to “Programa de merienda escolar” installed by the government since 1998.

The technique used for Skin Prick Test was the recommended according to the European standards [13].

Food allergy was diagnosed through oral food challenge at the allergy center under the supervision of a trained physician. An informed consent was obtained from each patient who recruited for OFC. Food suspected for allergy was strictly eliminated from the diet of individuals for 2 weeks prior to an OFC. Oral food and fluids were discontinued at least 12 h prior to OFC. During the OFC procedure, patients were monitored/supervised and regularly re-examined prior to each dose and at first signs of reaction. Total dose was divided into six incremental portions, where every next dose was double of the previous dose e.g. 1, 2, 4, 8 and 16 g of a solid food or 1, 2, 4, 8, 16 ml of liquid food. In case of suspected severe reaction a much smaller dose was decided.

In cases where patients’ complained of subjective symptoms (symptoms complained by the patients, i.e. throat or mouth itching, skin itching, or nausea) an observation period was allowed for the development of observable signs of an allergic reaction. However, in case no observable indications developed, OFC was continued. The results were considered “positive” until ‘moderate to severe objective clinical reactions’ occurred, such as respiratory, gastrointestinal, skin and cardiovascular symptoms. The challenge was stopped as soon as the observer was convinced that a reaction is occurring and medication was administered without delay.

### Statistical analyses

The clinical-epidemiological information was obtained from the electronic records of the databases of the computer unit of the Hospital María, was obtained in digital format and was later transferred to Excel where an ordering and selection was made and then analyzed in epiinfo 7.2.

### Ethical issues

The institutional approval of the Maria Specialty Hospital was obtained in accordance with the Declaration of Helsinki, preservation of the anonymity and privacy of research subject.

## Results

A total of 365 children were analyzed in this study, ranging in age from 1 to 18 years old with an average of 9.8 years. Skin prick testing with food extracts gave a positive result in 84 children to at least one food allergen (23%). The demographic characteristics are shown in Table 1.

**Table 1:** Characteristics Clinical epidemiological of the children in the immunology outpatient clinic

Variable	Sensitization to food allergen	
	Yes, n = 84 N (%)	No, n = 281 N (%)
Gender		
Female	48 (57.1)	122 (43.4)
Male	36 (42.9)	159 (56.6)
Pediatric stage		
Toddler (1–3 years)	9 (10.7)	13 (4.6)
Pre-school (4–6 years)	14 (16.7)	55 (19.6)
Childhood (7–11 years)	33 (39.3)	101 (35.9)
Early adolescence (12–14 years)	13 (15.5)	58 (20.6)
Late adolescence (15–18 years)	15 (17.9)	54 (19.2)
Diagnosis		
Multisystemic	61 (72.6)	199 (70.8)
Allergic rhinitis	11 (13.1)	38 (13.5)
Atopic dermatitis	5 (5.6)	14 (5.7)
Asthma	3 (3.6)	12 (4.3)
Urticaria	4 (4.8)	6 (2.1)
Atopic conjunctivitis	0 (0)	6 (2.1)
Food allergy	34 (9.3)	331 (90.7%)
Procedence		
Francisco Morazán	76 (88.6)	249 (90.5)
El Paraíso	4 (4.8)	7 (2.5)
Intibucá	0 (0)	10 (3.7)
Choluteca	0 (0)	4 (1.4)
Olancho	1 (1.2)	3 (1.1)
Comayagua	1 (1.2)	2 (0.7)
Atlántida	1 (1.2)	1 (0.4)
La Paz	1 (1.2)	1 (0.4)
Copan	0 (0)	1 (0.4)
Cortes	0 (0)	1 (0.4)
Santa Barbara	0 (0)	1 (0.4)
Valle	0 (0)	1 (0.4)

Females, 48 (57.1%) were the prevalent gender among the children that had a positive reaction to SPT. The majority of the sensitized children were in the 7–11 years old range (childhood), and 76 lived in Francisco Morazán (88.6%). Records provided showed the most part, 62 (72.6%) had a multisystem allergic disease in which they had at least two different allergic conditions that affected two different systems.

### Thirty-four (9.3%) patients were found to have food allergy and confirmed through OFC

Table 2 details SPT results for individual food allergen test, the rates of sensitization were as follows: milk 33 (9%), eggs 25 (6.9%), peanut 18 (4.9%), pork 16 (4.4%), fish and soybeans 15 (4.1%) each, chicken 12 (3.3%), Oat 11 (3%), wheat 10 (1.4%), Banana 8 (2.2%) and Rye 5 (1.4%).

**Table 2:** Prevalence of allergen sensitization in the immunology outpatient clinic

Allergen n = 365	Total	Positives %
Milk	33/365	9.0
Egg	25/365	6.9
Peanut	18/365	4.9
Pork	16/365	4.4
Fish	15/365	4.1
Soybeans	15/365	4.1
Chicken	12/365	3.3
Oat	11/365	3.0
Wheat	10/365	2.7
Beef	9/365	2.3
Banana	8/365	2.2
Rye	5/365	1.4

Sensitization to milk was the most frequent among food allergens. Thirty-three children had a positive SPT; 60.6% had a reaction to cow’s milks protein, while 27.3% had a positive response to casein, and 12.1% were to both allergens. Eggs were the next most common allergen, 44% had sensitization to egg whites, 28% to egg yolks and 28% to both egg whites and yolk.

**Table 3:** Prevalence of confirmed food allergy in sensitized patients through oral food challenge

Allergen	OFC participants, n = 365 N (%)
Animal protein	
Milk	22 (6.0)
Egg	19 (5.2)
Pork	6 (1.6)
Chicken	5 (1.4)
Beef	4 (1.1)
Fish	3 (0.8)
Plant food	
Wheat	7 (1.9)
Peanuts	3 (0.8)
Fish	3 (0.8)
Soybeans	2 (0.5)
Banana	1 (0.3)
Rye	1 (0.3)
Oat	ND

*ND* not done

Out of 365 children that visited the immunology outpatient clinic. 97.3% did not report history of food allergy, all patients were screened through SPT to determine sensitivity and open OFC challenge was done to patients that required a diagnosis.

Food specific OFC results show that the milk is the most prevalent food allergen affecting 6% of the total patients; meanwhile and 5.2% correspond to hen’s egg allergen prevalence in the OFC trial group.

Wheat was the frequent plant food allergen with a prevalence of 1.9%, as shown in Table 3. Four patients had history of anaphylaxis; written emergency actions plans were given to parents to help them recognize early signs and treatment.

Nineteen children were diagnosed with atopic dermatitis and were classified according to the SCORAD (Scoring atopic dermatitis) punctuations, as seen in Table 4. Sixteen (84.2%) children had both, poly-sensitization and atopic dermatitis. Fourteen patients were multisystemic, or had another atopic disease as comorbidity.

**Table 4:** Relationship between patients with eczema and degree of sensitization

Patients with eczema	Type of sensitization		
SCORAD	Mono-sensitization, n = 3 N (%)	Poly-sensitization, n = 16 N (%)	Total n = 19 N (%)
Mild (< 20)	0 (0)	2 (100.0)	2 (10.5)
Moderate (15–40)	2 (20)	8 (80.0)	10 (52.6)
Severe (> 40)	1 (14.3)	6 (85.7)	7 (36.8)

Children with eczema were younger, with a median age of 7. The prevalence of poly-sensitization in this group was 84.2%. Based on SCORAD score eczema was evaluated as mild in 10.5%, moderate was the most frequent 52.6% followed by severe in 36.8%, the mean SCORAD score is 32.6 (range of: 13–48.7).

Among 84 food-sensitized children, obesity was the most common comorbidity found in 14 (16.7%) children followed by GERD in 9 (10.7%), conduct disorders and acne in 6 for each (7.1%) as seen in Table 5.

**Table 5:** Comorbidities found in the food sensitized children in the outpatient clinic

Comorbidities	Total	Positives %
Obesity	14/84	16.7
GERD	9/84	10.7
Conduct disorders	6/84	7.1
Acne	6/84	7.1
Gastritis	3/84	3.6
Low weight	3/84	3.6
Thyroiditis	3/84	3.6
Depression	3/84	3.6
Headaches	2/84	2.4
Arthritis	2/84	2.4
Epilepsy	1/84	0.8

## Discussion

This study determined the prevalence of food sensitization through SPT among 365 children with allergic diseases. The age of participants were in the range from 1 to 18 years, with an average of 9.8 years. The individual prevalence of food allergen sensitization type was different in our study, which may be due to cultural differences in food habits, although we cannot exclude the possibility that the lower size of our sample in comparison with other studies might have influenced the results. In spite of the fact we performed open food challenges in some of the patients, it was not possible to perform double blind placebo-controlled food challenges, which are regarded as the “gold standard” for the final diagnosis of food allergies. This was a weakness of our methodology.

Sensitization to at least one food allergen was found in 23%, Similar sensitization rates had been found in similar studies in children in Germany 20% [16] and Iran has reported higher rates 40% in previous studies [17]. Thirty-four (9.3%) patients in this study were found to have food allergy confirmed through OFC. The types of foods most frequently implicated in our study, are included: milk, egg, peanut, wheat, and soybeans, fish.

Food allergy is an adverse immune response to a food. It is seen in 2–8% of children, mostly infants and small children, according to the data from the USA and Western Europe, the leading causes of food allergy in childhood are cow’s milk proteins (2.0–3.5%), eggs (1.3–3.2%) peanuts (0.6–1.3%), fish (0.4–0.6%) and tree nuts (0.2%) [18]. In Mexico, the Mexipreval study conducted in the year 2013–2014 reported that the highest frequency of suspected allergic reactions could be related to milk (44.5%), fruit (25.4%), egg (21.8%), cereals 19.6%), seafood (13.6%), vegetables (10.3%), nuts (9.2%) and fish (8.2%) [19].

Cow’s milk and eggs are one of the most important animal food allergens; therefore we evaluated the sensitization rate in this study. These patients demonstrated a 9% sensitization to milk allergens, similar findings were reported in children in Brazil 9.1% [20], 3.5% China [21], and 5.6% Australia [22]. Cow’s milk allergy (CMA) was confirmed in 6% of the sensitized patients, this is a lower rate compared to a study in United States were a prevalence of 19.9% was found [23]. After CMA, hen’s egg allergy is the second most common food allergy in infants and young children. Worldwide records estimate 31% egg allergy in infants [24] and 0–18% in children of all ages [25].

Animal protein sensitization to meats has been poorly described. A study in Mexican children described 13.79% had sensitivity to pork, 8.62% to chicken and 1.72% to beef meat [26]. Data in the present work shows sensitivity prevalence of 4.4% to pork, 3.3% to chicken and 2.5% to beef; this results are similar to what has previously been described. We found no previous data that describes animal meat proteins allergy in children, however we report a 1.6% pork meat allergy, 1.4% to chicken allergy, and 1.1% to beef allergy. This may be due to tick exposure. Humans come into contacts with ticks during warmer weather, consistent with Honduran climate. Allergies that develop to these tick bites result in IgE that targets the carbohydrate alpha-gal. Alpha- gal is present in mammalian meat, and is responsible for many cases of mammalian meat allergy. In addition, that tick bites represent the most important cause of alpha-gal allergies by inducing specific antibodies [27].

Fish sensitization is 4.1% prevalence. Fish is a major source of dietary protein, especially in coastal areas, like Honduras. Higher sensitization for this allergen was found in Egyptian children with a prevalence of 13.8% [10]. Fish allergy prevalence confirmed via food challenge in our study was 0.8%. This is a lower rate compared to a study in the United States were sensitization rates were higher in Hispanic children for fish allergy with a prevalence of 16.2% [28]. One possibility for this difference is in spite of the country having two coasts, highly allergenic fish (salmon, swordfish, catfish and rainbow trout) are not consumed.

Wheat sensitivity via SPT was 2.7%, higher rates have being described in children in Iran were a prevalence of 18.3% was found [29]. 1.9% of the sensitized children had a confirmed allergy. The prevalence of clinically relevant of wheat allergy is not well established, but it is estimated to be less than 0.5–1% with most children outgrowing it by age 16 [30].

Sensitization to peanuts was the third frequent allergen in the study group, 4.9%. These data closely match with earlier reports in Latin America were 5.27–9.4% rates were established [31–33]. The United States reports a higher prevalence, 13.04% [34]. Peanut allergy in our study was confirmed in 0.8% of the participants, similar results were found in Canada where a prevalence of 1.5% was reported [35].

Soy protein is one major food components of the national program for scholar’s meals (programa nacional de merienda escolar) in Honduras. A study in children in Chile reported a 1.3% positive SPT reaction; our study population prevalence was 4.1%, similar with previous data [36]. Soy allergy is not common as cow’s milk allergy in children. Data in the present work shows 0.5% suffering from soy allergy; this is in line with previous population-based studies, two European cohorts pointed rates varying up to 0.7% for children in double-blind placebo-controlled food challenge [37].

Banana, rye and oat are uncommon food allergens. Banana is one of the frequent fruits consumed by Honduran children. We found 2.2% sensitivity to banana in the study population. This result is similar to a study in Egyptian children that showed 7.5% had a positive SPT to the same allergen [38]. Eleven (3.0%) patients visiting the outpatient clinic were sensitized to oat. The prevalence found in this study is significantly lower when compared to a study in atopic children in France where 19.2% had a positive SPT reaction to this food allergen [39]. Rye sensitivity in this study was 1.4%. Banana and oat allergy were confirmed via food challenge in our study in 0.3% of the children respectively. Patients sensitized to oat did not have oral food challenge performed.

Patients with atopic dermatitis were more likely to be poly-sensitized. Poly-sensitization and a moderate SCORAD was found in 50% of the children and coexistence between poly-sensitization and severe score was found in 31.6% of the patients. Our results are similar to a study in Netherlands that showed that children with AD faced a considerably higher risk of being sensitized to multiple allergens. Poly-sensitization was more than five times as common in children with AD than in those without [40].

A relationship between obesity and atopy has been described before, but not consistently, and food sensitization has rarely been examined in relation to obesity. This study found that 14 (16.7%) patients that had food sensitization also had obesity. Many studies have shown that children with restrictive eating problems as FA, frequently affects quality of life, higher levels of stress and anxiety [41]; the related disorders include changes in mood, deterioration in cognitive function and in school and work performance, externalizing and oppositional behaviors [42, 43].

Food allergy remains an important health concern due to increasing prevalence worldwide, cow’s milk allergy is the most prevalent, followed by hens egg allergy and wheat. Milk, egg and peanut were the frequent allergens that patients were sensitized to. This patient population had a higher rate of confirmed mammalian meat allergy compared to previous reports from North America.

## Abbreviations

FA: food allergy
SPT: skin prick test
CMA: cow’s milk allergy
